# Metagenomic Analysis of Taxa Associated with *Lutzomyia longipalpis*, Vector of Visceral Leishmaniasis, Using an Unbiased High-Throughput Approach

**DOI:** 10.1371/journal.pntd.0001304

**Published:** 2011-09-06

**Authors:** Christina B. McCarthy, Luis A. Diambra, Rolando V. Rivera Pomar

**Affiliations:** 1 Centro Regional de Estudios Genómicos, Universidad Nacional de La Plata, Florencio Varela, Buenos Aires, Argentina; 2 Departamento de Ciencias Básicas y Experimentales, Universidad Nacional del Noroeste de la Provincia de Buenos Aires, Pergamino, Buenos Aires, Argentina; IRD/CIRDES, Burkina Faso

## Abstract

**Background:**

Leishmaniasis is one of the most diverse and complex of all vector-borne diseases worldwide. It is caused by parasites of the genus *Leishmania*, obligate intramacrophage protists characterised by diversity and complexity. Its most severe form is visceral leishmaniasis (VL), a systemic disease that is fatal if left untreated. In Latin America VL is caused by *Leishmania infantum chagasi* and transmitted by *Lutzomyia longipalpis*. This phlebotomine sandfly is only found in the New World, from Mexico to Argentina. In South America, migration and urbanisation have largely contributed to the increase of VL as a public health problem. Moreover, the first VL outbreak was recently reported in Argentina, which has already caused 7 deaths and 83 reported cases.

**Methodology/Principal Findings:**

An inventory of the microbiota associated with insect vectors, especially of wild specimens, would aid in the development of novel strategies for controlling insect vectors. Given the recent VL outbreak in Argentina and the compelling need to develop appropriate control strategies, this study focused on wild male and female *Lu. longipalpis* from an Argentine endemic (Posadas, Misiones) and a Brazilian non-endemic (Lapinha Cave, Minas Gerais) VL location. Previous studies on wild and laboratory reared female *Lu. longipalpis* have described gut bacteria using standard bacteriological methods. In this study, total RNA was extracted from the insects and submitted to high-throughput pyrosequencing. The analysis revealed the presence of sequences from bacteria, fungi, protist parasites, plants and metazoans.

**Conclusions/Significance:**

This is the first time an unbiased and comprehensive metagenomic approach has been used to survey taxa associated with an infectious disease vector. The identification of gregarines suggested they are a possible efficient control method under natural conditions. Ongoing studies are determining the significance of the associated taxa found in this study in a greater number of adult male and female *Lu. longipalpis* samples from endemic and non-endemic locations. A particular emphasis is being given to those species involved in the biological control of this vector and to the etiologic agents of animal and plant diseases.

## Author Summary

Leishmaniasis is a vector-borne disease with a complex ecology and epidemiology. It has three main clinical forms of which visceral leishmaniasis (VL) is the most severe, as it is fatal if untreated. It is caused by a protist parasite, *Leishmania* spp., and is transmitted to humans by phlebotomine sandflies. The best method to interrupt any vector-borne disease is to reduce man-vector contact. Vector-targeted strategies are particularly attractive because the vectorial capacity to transmit infectious diseases to humans is proportional to vector density and, in an exponential way, to vector survival. Biological control is an effective means of reducing or mitigating pests through the use of natural enemies and is more environmentally friendly than traditional insecticide treatments. Nevertheless, there is very scanty information on the biological control of sandflies and their potential control agents. In this context, a detailed knowledge of the microorganisms that are associated with these vectors would aid in the development of novel strategies for controlling them. This is the first study to survey the taxa associated with leishmaniasis vectors and, more importantly, with any infectious disease vector, using an unbiased and high-throughput approach.

## Introduction

Leishmaniasis is a vector-borne neglected infectious disease of worldwide incidence and its most severe clinical form is visceral leishmaniasis (VL). Each year VL causes an estimated 500,000 new cases and more than 59,000 deaths [1], a death toll that is only surpassed by malaria among the parasitic diseases [2]. Furthermore, both figures are approximations since VL is frequently not recognized or not reported [3]–[4]. Leishmaniasis is transmitted through the bite of two phlebotomine sandfly genera, *Phlebotomus* in the Old World and *Lutzomyia* in the New World. In Latin America VL is caused by *Leishmania infantum chagasi* and transmitted by *Lutzomyia longipalpis* [5]. This phlebotomine sandfly is only found in the New World, with a wide distribution from Mexico to Argentina [6]. The geographical distribution of leishmaniasis has undoubtedly expanded and is now being reported in areas that were previously non-endemic. The worldwide phenomenon of urbanisation, closely related to the sharp increase in migration, is one of the major risk factors that is making leishmaniasis a growing public health concern for many countries around the world [7] and Argentina is not an exception. Between 1925 and 1989 only 14 leishmaniasis human cases were reported in Argentina and none was attributed to *Le. chagasi*. Moreover, there were only two isolated reports of *Lu. longipalpis* (in 1953 and 2000) which were not associated with VL [8]. Nevertheless, this situation has changed dramatically, mostly due to an indiscriminate advance of urbanisation, and the first Argentine VL outbreak was recently reported [9]. From 2006 to date the morbidity and mortality toll of this disease have amounted to 83 human cases (35% corresponding to children under ten years of age), 7 deaths and more than 7,000 infected dogs (National Health Surveillance System, Epidemiology Bureau, National Ministry of Health, Argentina).

In the natural environment, phlebotomine larvae feed on organic matter from soil [10], while adults from both sexes feed on sugars from plant sources [11]–[12]. Only female adults need blood to obtain necessary proteins for the development of their eggs [5]. It is widely accepted that many insects derive their microbiota from the surrounding environment, such as the phylloplane of food plants or the skin of the animal host, and although the degree of persistence of strains of the ingested species is unknown, these microorganisms can influence the insect life cycle [13]. To comprehensively understand the biology of insects, microorganisms must be considered as a very important component of the ecological system [14]. Moreover, an inventory of the associated microbiota of phlebotomine sandflies, especially of wild specimens, would aid in understanding the annual and regional variations recorded for this disease [15] and in the development of novel strategies for controlling these vectors, among others [13]. One serious obstacle for the biological control of VL sandfly vectors is that their precise breeding sites are poorly known. Furthermore, its practical application seems to be limited to the adult VL vector stage [16] because, as *Lu. longipalpis* larvae appear to be thinly dispersed [17], this complicates the employment of biolarvicides in the field.

There is scanty information on the microbial colonisation of *Lu. longipalpis* and it is not yet clear if they possess an indigenous community. Previously, midgut bacteria were examined from wild and laboratory reared *Lu. longipalpis* populations [18]–[20] which showed a predominance of Gram negative bacteria. Various genera found ubiquitously in the environment (water, soil and debris) were identified in these studies, including *Acinetobacter*, *Serratia*, *Pseudomonas*, *Stenotrophomonas*, *Flavimonas* and *Enterobacter*. These bacteria have also been found associated with the gut of several other insects [21]–[24], suggesting they are a part of the natural or transient microbiota. Prior studies on guts and malpighian tubes from wild *P. papatasi* and *P. tobbi* showed a high incidence of mycoses which were similar to *Aspergillus sclerotiorum* and *Saccharomyces cerevisiae* [25]. Various types of virus have also been found infecting phlebotomine sandflies [26], including *Vesiculovirus* [27]–[28] and Cytoplasmic Polyhedrosis Virus [29]. Furthermore, in addition to *Leishmania*, *Trypanosoma*, *Endotrypanum* and possibly other trypanosomatids [30], neotropical sandflies may harbour other parasites including microsporidians [31]–[32], gregarines [33]–[36], some *Plasmodium* spp. that parasitise lizards [37] and nematodes [38]–[41]. Nevertheless, there is little information on the pathological effects these parasites may produce in their sandfly hosts.

Metagenomics facilitates the culture-independent analysis of microbial communities [42], an approach which does not require prior assumptions about the composition of the target community. Metagenomic sequencing of communities containing eukaryotes, in particular protists, is mostly cost-prohibitive because of their enormous genome sizes and low gene coding densities [43]. Nevertheless, from an ecological perspective, excluding eukaryotes from a metagenomic analysis compromises the ability to assess the microbial community in its entirety. A possible approach to bypass the problem of large amounts of non-coding eukaryotic sequence data consists in obtaining molecular data at the RNA level. Given the recent VL outbreak in Argentina and with the ultimate goal of identifying possible biological control agents, this study used unbiased high-throughput pyrosequencing technology [44] to compare the diversity of the taxonomic groups associated with wild male and female adult *Lu. longipalpis* from endemic (Posadas, Misiones) and non-endemic (Lapinha Cave, Minas Gerais) VL locations in Argentina and Brazil, respectively. As in this study phlebotomine sandflies were considered environmental samples, the term “associated with” was used here in its broadest sense, referring to a wide variety of possible interactions ranging from casual associations due to random environmental contact (*e.g.*, plant pathogenic fungi spores adhering to the hairy surface of the sandflies when sugar-feeding on plants) to closer pathogenic or symbiotic interactions (*e.g.*, protists that parasitise phlebotomines or permanent gut microbiota, respectively). This analysis revealed the presence of sequences from bacteria, fungi, protists, plants and metazoans.

## Methods

### Ethics statement

This study was carried out in strict accordance with the recommendations in the Manual for the Use of Animals/FIOCRUZ (Manual de Utilização de Animais/FIOCRUZ) of Fundação Oswaldo Cruz, FIOCRUZ, Ministry of Health of Brazil (National decree Nr 3,179). The protocol was approved by the Ethics Committee for the Use of Animals of the Fundação Oswaldo Cruz - FIOCRUZ, Ministry of Health of Brazil (Nr 242/99).

### Field sampling and specimen preparation

*Lu. longipalpis* specimens from the non-endemic VL location, Lapinha Cave (Minas Gerais, Brazil), situated in the Sumidouro National Public Park, were kindly provided by Dr. Paulo Pimenta (Laboratory of Medical Entomology, Centro de Pesquisas René Rachou, Fundação Oswaldo Cruz, FIOCRUZ). Sandflies from this location were chosen as reference because they have been extensively studied. *Lu. longipalpis* specimens from the endemic VL location, Posadas (Misiones, Argentina), where they occur in high density, were kindly provided by Dr. María Soledad Santini and Mr. Enrique Adolfo Sandoval (Research Network for Leishmaniasis in Argentina, REDILA, and Posadas Municipality Quality of Life Department).

Captures were made using CDC light traps [45] on the 15th and 26th of May 2009 in the Lapinha Cave and in Posadas, respectively. The Lapinha Cave (S19 33 42.42 W43 57 34.96) is a network of interconnected caves located in a vast tropical savanna ecoregion called *cerrado*, characterised by great plant and animal biodiversity. The trap was left 50–80 cm above ground level in an external small annex cave (2 mt long) where a chicken was kept to attract the sandflies and as a source of food (see Table 1 for a detailed description of the site). Posadas, the densely populated capital city of the province of Misiones, is located in the subtropical fields and grasslands ecoregion. In the Posadas area this ecoregion contacts the Paranaense forest and has a savanna-type landscape. The trap was installed in the peridomicile of a worst-case scenario homestead (domestic animals, dense vegetation, nearby spring) (S27 23.266 W55 53.403) (see Table 1 for a detailed description of the site).

**Table 1:** Ecological description of the EVL (Posadas, Argentina) and NEVL (Lapinha Cave, Brazil) sampling site locations.

	Ecological description		
Sampling site	Animals	Plants	Others
**Posadas**	**Domestic animals present in the homestead:** *Canis lupus familiaris* (dog); *Felis catus* (cat); *Gallus gallus* (chicken)	**Homestead plants:** *Myrtus communis* (common myrtle); *Citrus x sinensis* (orange tree); *Citrus x limon* (lemon tree); *Delonix regia* (flame tree); *Punica granatum* (pomegranate); *Jacaranda* spp.; *Persea americana* (avocado); *Ficus carica* (fig tree)	Family homestead situated in a densely populated urban area with a spring of water, dense vegetation and abundant organic matter produced by domestic animals and humans.
**Lapinha Cave**	**Animal species confirmed at the time of sampling:** *Gallus gallus* (chicken), *Homo sapiens* (human). **Other animal species found in the area:** Mammals: Dasypodidae (armadillos); *Leopardus tigrinus* (tiger cat); *Lontra* spp. *(otter)*; *Tamandua tetradactyla* (tamanduá-de-colete). Reptiles: *Crotalus* spp.; *Sistrurus* spp.; *Bothrops* spp.; Boidae. Birds: *Buteogallus* spp.; *Buteo* spp.; *Leucopternis* spp.; *Dendrocygna viduata* (white-faced whistling duck); *Phalacrocorax brasilianus* (Neotropic cormorant)	**Plant species found in the area:** *Tabebuia chrysantha* (yellow Ipê); *Tabebuia serratifolia* (yellow lapacho); *Lithraea molleoides* (aruera); *Campomanesia pubescens* (guabiroba); *Hymenaea stigonocarpa* (jatobá do campo)	Cave situated in a damp environment, with organic matter produced by animals (chicken and bats, among others) and a nearby lake. Located in the *cerrado* ecoregion which is characterised by a community of trees and large shrubs, usually 2–8 m in height, belonging to many species and producing 10–60% coverage, with a well-developed grassy ground layer between. The ground layer is usually about 60 cm tall and consists of many species of grasses and sedges mixed with a great diversity of forbs, amongst which Leguminosae, Compositae, Myrtaceae and Rubiaceae families are the most important [88].

Posadas, the capital city of the province of Misiones, is large, densely populated and located in the Argentine fields and grasslands ecoregion. The Lapinha Cave is a tourist attraction site located in the Sumidouro National Public Park which is part of the Brazilian *cerrado* ecoregion.

Sandflies were transported alive in a nylon cage to the corresponding laboratories in Belo Horizonte (Minas Gerais) and Posadas (Misiones) and no mortality was registered on arrival. Other insect species were captured together with the sandflies including hymenopterans, lepidopterans and mosquitoes. Sandflies were killed at low temperature, identified and separated according to sex, and stored alternatively in Tri-Reagent (Molecular Research Center Inc., Cincinnati, OH) or RNAlater® (Qiagen). A total of four groups of 100 sandflies each, two per location, were separated and named according to: SS1, females from the Endemic VL location (EVL females); SS2, EVL males; PP1, females from the Non-Endemic VL location (NEVL females); and PP2, NEVL males.

### Sample preparation

Individual samples were ground in Tri-Reagent (Molecular Research Center Inc., Cincinnati, OH) with a Teflon pestle and total RNA was immediately extracted, according to the manufacturer's instructions. Total RNA was amplified using a modified sequence-independent amplification protocol [46]. Briefly, M-MuLV Reverse Transcriptase (Fermentas, Vilnius, Lithuania) was used for a first-strand reverse transcription which was initiated with a random octamer linked to a specific primer sequence (5′-GTT TCC CAG TAG GTC TCN NNN NNN N-3′) [47]. cDNA was then amplified with the Expand Long Template PCR System (Roche) using a 1∶9 mixture of the above primer and a primer targeting the specific primer sequence (5′-CGC CGT TTC CCA GTA GGT CTC-3′) [48]. The following profile was used: initial denaturation cycle at 94°C for 2 minutes; five low stringency cycles with denaturation at 94°C for 30 seconds, 25°C for 30 seconds and 68°C for 6 minutes, were followed by 30 cycles at 94°C for 30 seconds, 55°C for 30 seconds and 68°C for 6 minutes and a final extension cycle at 68°C for 5 minutes. Pooled samples were submitted for high-throughput pyrosequencing (Macrogen Inc., Korea).

### Sequence accession numbers

Reads were submitted to the NCBI Sequence Read Archive (SRA) (submission SRA026595) under accessions SRR089611 (adult EVL female *Lu. longipalpis*; Posadas, Misiones, Argentina; SS1), SRR089612 (adult EVL male *Lu. longipalpis*; Posadas, Misiones, Argentina; SS2), SRR089613 (adult NEVL female *Lu. longipalpis*; Lapinha Cave, Minas Gerais, Brazil; PP1) and SRR089614 (adult NEVL male *Lu. longipalpis*; Lapinha Cave, Minas Gerais, Brazil; PP2).

### Sequence analysis

Reads ranged in size from approximately 100 to 1200 base pairs (bp) (350 bp average). Raw sequence reads were trimmed to remove sequences derived from the amplification primer. With the purpose of reducing database search efforts and improving the homology detection sensitivity [49], Cd-hit [50] was used to generate non-redundant nucleotide datasets but these represented less than 1% in every case (data not shown). For this reason, singlet sequences were used for the nucleotide database search. Non-redundant (nt) and non-human, non-mouse ESTs (est-others) NCBI databases last modified on 23/04/10 and 25/04/10, respectively, were downloaded locally (ftp://ftp.ncbi.nlm.nih.gov/blast/db/). After trimming, singlet sequences were compared to these databases using BLASTN (nucleotide homology) [51], with a 1e-50 cutoff E-value. The resulting BLAST alignments were analysed and classified according to their taxonomical hierarchies using custom applications written in Mathematica (Wolfram Mathematica 7; available upon request). 16S sequences were confirmed by alignment to type-species 16S rRNA sequences from the Ribosomal Database Project (http://rdp.cme.msu.edu/) [52]–[53]. Hits for every taxon were individually revised and confirmed and only those which showed unequivocal results were included in the final analysis. Fisher's Exact Test [54] (p<0.05) was used to establish the significance of sequences in the different samples using a custom application written in Mathematica (Wolfram Mathematica 7; available upon request).

## Results

The vast majority of reads obtained for each sample corresponded to *Lu. longipalpis* sequences (∼85%) and an important fraction showed no significant hits in the homology searches against the different databases (∼14%). Hits which corresponded to taxa other than *Lu. longipalpis* represented less than 0.2% of the total reads.

**Figure 1:**
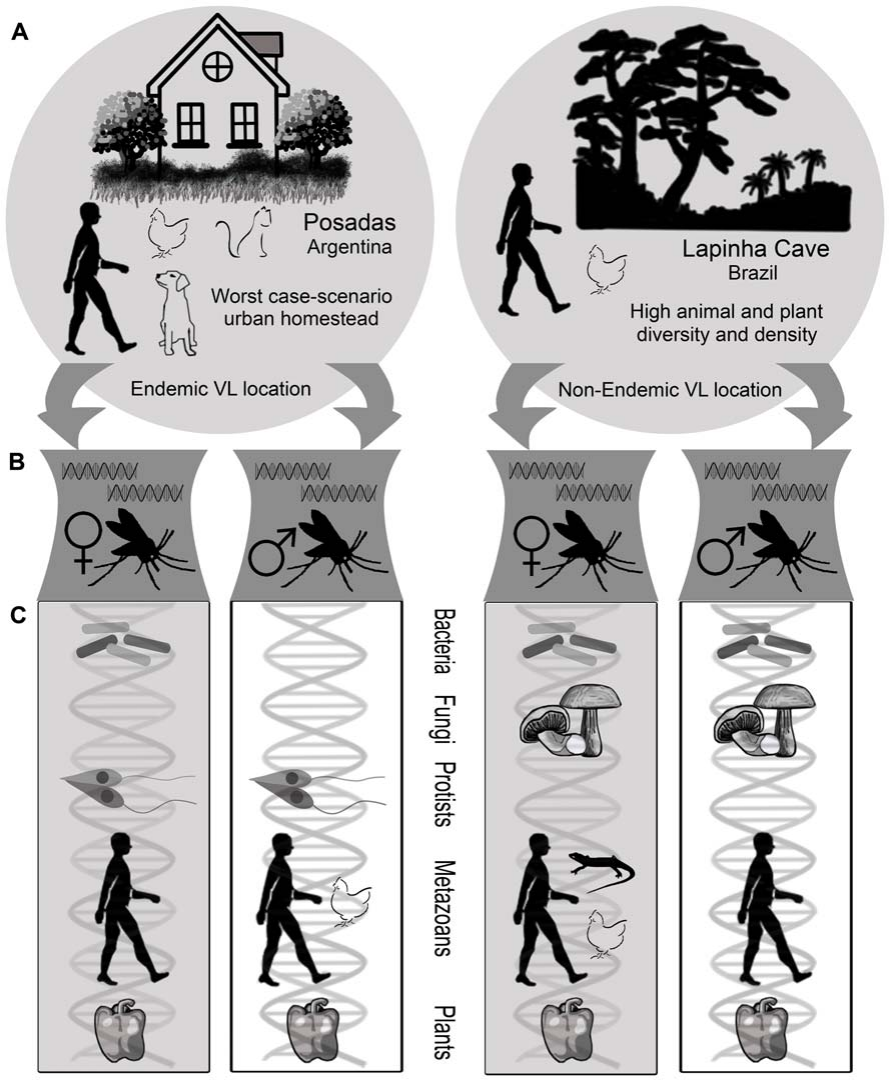
VL vectors as environmental samples: taxa identified in phlebotomine sandflies considering sampling site conditions. This figure summarises and associates sampling site characteristics with taxa identified in male and female adult *Lu. longipalpis* from both locations. In all cases figures are only schematic and not an exact representation of either the sampling sites, phlebotomine sandflies or identified taxa. A) Shows the most significant ecological characteristics of both phlebotomine capture site locations in Argentina and Brazil: Posadas and Lapinha Cave, respectively. Only those animal species confirmed at the time of sampling were represented schematically (Table 1 includes a detailed list of animal and plant species in both locations). (B) Total RNA was extracted from male and female adult *Lu. longipalpis* specimens and amplified using sequence independent amplification (see Methods section). (C) Shows the different taxa identified by sequence homology in all four samples (bacteria, fungi, protists, metazoans and plants). Taxa are represented schematically and the particular species identified for each taxonomical group are not shown, except in the case of metazoans (see Figure 2 for a detailed list). Grey rectangular boxes group taxa found in female adult *Lu. longipalpis*. White rectangular boxes group taxa found in male adult *Lu. longipalpis*. VL: Visceral Leishmaniasis.

**Figure 2:**
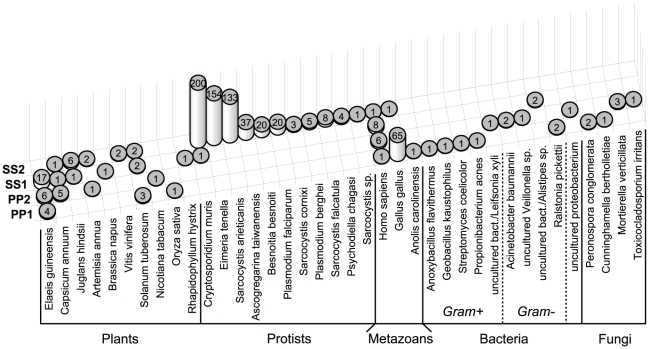
Taxa associated with wild adult male and female *Lu. longipalpis* specimens analysed in this study. Results for all the samples were integrated and summarised in this figure, which indicates all the taxonomical groups that were identified, the species that were found for each taxon and the number of sequences for each species. Solid black lines group the different taxa (plants, protists, metazoans, bacteria and fungi). For bacteria, the dotted black lines separate and differentiate Gram+ and Gram- rods. The different species that were found are named beneath each column. The number of sequences for each species is indicated on the top part of each cylinder. SS1: adult females from the Endemic VL location (EVL); SS2: adult EVL males; PP1: adult females from the Non-Endemic VL location (NEVL); PP2: adult NEVL males.

Results for each taxon were organised separately in this section. Figure 1 emphasises the treatment of these vectors as environmental samples. It shows an overview of the workflow used in this study, summarising and associating information on the sampling sites and on the taxa identified by sequence homology in each adult *Lu. longipalpis* sample. A detailed ecological description of both sampling sites is given in Table 1. Figure 2 integrates and summarises results for all the samples, indicating the taxa that were identified in each case, the species that were found for each taxon and the number of sequences for each species. Table S1 lists all the reads that showed significant hits and a brief description of each hit.

### Bacteria

BLASTN analysis of the high-throughput sequencing data identified bacteria in females from both locations (SS1 and PP1) and in NEVL males (PP2) (Figures 1 and 2). Bacteria were identified mostly by homology to completely sequenced bacterial genomes (7 reads), followed by rRNA genes (4 reads) and lastly to plasmid sequences (2 reads) (Table S1). Ten different bacterial types were identified, six of which showed homology at the species level (five to genomic sequences and one to plasmid sequences) and four to diverse uncultured environmental samples (three to 16S rRNA genes and one to genomic sequences). The bacterial composition was different and unique in every case and included sequences from *Ralstonia pickettii*, *Anoxybacillus flavithermus*, *Geobacillus kaustophilus*, *Streptomyces coelicolor*, *Propionibacterium acnes*, *Acinetobacter baumannii*, uncultured *Veillonella* sp. and uncultured bacterium clones isolated from environmental samples (cow faeces, wetland soil and water) (Figure 2; Table S1).

The totality of identified bacteria showed a predominance of Gram negative rods (53.8%, 7 reads) and a significant proportion of Gram positive bacteria (38.5%, 5 reads), in accordance with previous studies [18]–[20], [55]. Of all the bacterial sequences that were identified in this study, only *A. baumannii*, which was found in NEVL males (PP2), had been previously identified in adult female *Lu. longipalpis*. This species had been isolated from female laboratory reared specimens from the same non-endemic VL location (Lapinha Cave) [20] and from wild female specimens from endemic VL locations in Brazil (Jacobina, Bahia, and São Luís, Maranhão) [18].

### Fungi

Fungi were only found in NEVL males and females (PP1 and PP2) (Figure 1). A total of four different species was identified by homology to rRNA genes (Figure 2; Table S1). These differed between males and females and have not been found to date associated with phlebotomines. The identified species included *Peronospora conglomerata*, *Cunninghamella bertholletiae*, *Mortierella verticillata* and *Toxicocladosporium irritans* (Figure 2; Table S1).

### Protists

Protist sequences were only identified in EVL female and male specimens (SS1 and SS2) (Figure 1), of which the vast majority (99.8%) were found in males (Figure 2; Table S1). Protists were identified by homology to sequenced rRNA genes (360 reads, 61.4%), cDNA (208 reads, 35.5%) and chromosomal sequences (18 reads, 3.1%) (Table S1). Ten species and one genus of apicomplexan parasites were identified that parasitise Diptera (*Ascogregarina taiwanensis*, *Psychodiella chagasi*), birds (*Eimeria tenella*, *Sarcocystis falcatula*, *Sarcocystis cornixi*), mammals (*Cryptosporidium muris*, *Sarcocystis arieticanis*, *Besnoitia besnoiti*, *Plasmodium falciparum*, *Plasmodium berghei*) and reptiles, birds and mammals (*Sarcocystis* sp.) (Figure 2; Table S1). Most of the apicomplexan sequences (64.8%, 379 reads) were homologous to the mammalian parasites *C.muris*, *S. arieticanis*, *B. besnoiti*, *P. falciparum* and *P. berghei*. Of these, more than half (53.6%, 203 reads) were homologous to published cDNA libraries and the rest to rRNA genes (41.7%, 158 reads) and chromosomal DNA (4.7%, 18 reads). The second most numerous group of apicomplexan sequences was homologous to the avian parasites *E. tenella*, *S. cornixi* and *S. falcatula* rRNA genes (28.2%, 165 reads) and the rest were homologous to the dipteran parasites *A. taiwanensis* and *P. chagasi* rRNA genes (7%, 41 reads).

### Metazoans

Metazoan sequences (mammals, birds and reptiles) were also found in all the samples and included *Homo sapiens*, *Gallus gallus* and *Anolis carolinensis* (Figures 1 and 2). *Homo sapiens* was identified by homology to genomic chromosomal sequences (16 reads). *Gallus gallus* was identified by homology to genomic chromosomal sequences (46 reads), cDNA (18 reads) and rRNA genes (1 read). *Anolis carolinensis* was identified by homology to cDNA (1 read) (Table S1). Human sequences were found in males and females from both locations, whereas chicken sequences were found in NEVL females (PP1) and EVL males (SS2) and lizard sequences were only found in NEVL females (PP1) (Figures 1 and 2).

### Plants

A total of ten different plant species were identified in males and females from both locations, namely *Elaeis guineensis*, *Capsicum annuum*, *Juglans hindsii*, *Artemisia annua*, *Brassica napus*, *Vitis vinifera*, *Solanum tuberosum*, *Nicotiana tabacum*, *Oryza sativa* and *Rhapidophyllum hystrix* (Figures 1 and 2). All plant sequences were identified by homology to cDNA libraries (56 reads), except for *R. hystrix* which was identified by homology to rRNA genes (1 read) (Table S1). EVL males and females showed a greater number of species (5 and 6 species, respectively), followed by NEVL males (4 species) and lastly NEVL females (3 species) (Figure 2). However, these differences were significant (p<0.05) only between females from both locations (Table 2). EVL females showed the highest number of plant sequences (23 reads), followed by EVL and NEVL males (13 reads) and lastly NEVL females (8 reads) (Figure 2). The only case in which these differences were not significant (p<0.05) was between EVL females and NEVL males (Table 3).

**Table 2:** Statistical analysis of the number of plant species between samples (Fisher's Exact Test; p<0.05).

	SS1	SS2	PP1	PP2
**Total species**	9	18	13	11
**Plant species**	6	5	3	4
**SS1**	NA	0.0552015	0.0482972	0.150036
**SS2**	0.0552015	NA	0.310627	0.28232
**PP1**	0.0482972	0.310627	NA	0.272693
**PP2**	0.150036	0.28232	0.272693	NA

The total number of associated taxa (total species) that were found for each sample and the total number of plant species are indicated for every sample. NA: not applicable; SS1: adult females from Endemic VL location (EVL); SS2: adult EVL males; PP1: adult females from Non-Endemic VL location (NEVL); PP2: adult NEVL males.

**Table 3:** Statistical analysis of the number of plant sequences between samples (Fisher's Exact Test; p<0.05).

	SS1	SS2	PP1	PP2
**Total sequences**	34	600	84	28
**Plant sequences**	23	13	8	13
**SS1**	NA	7.18638×10^−25^	4.61455×10^−10^	0.0510662
**SS2**	7.18638×10^−25^	NA	0.00162601	8.40853×10^−13^
**PP1**	4.61455×10^−10^	0.00162601	NA	5.71033×10^−05^
**PP2**	0.0510662	8.40853×10^−13^	5.71033×10^−05^	NA

The total number of reads of associated taxa that showed significant hits (total sequences) and the total number of plant sequences are indicated for every sample. NA: not applicable; SS1: adult females from Endemic VL location (EVL); SS2: adult EVL males; PP1: adult females from Non-Endemic VL location (NEVL); PP2: adult NEVL males.

*C. annuum* (bell pepper) was found in EVL males and females and in NEVL males. *E. guineensis* (African oil palm) was found in EVL females and NEVL males and females. *S. tuberosum* (potato) was found in EVL males and females and in NEVL females. *J. hindsii* (Northern California walnut) was found in EVL males and females and *A. annua* (sweet wormwood) was found in males from both locations. *R. hystrix* (needle palm) and *B. napus* (rapeseed) were only found in EVL females and *V. vinifera* (grapevine) was only found in EVL males. *O. sativa* (rice) was only found in NEVL females and *N. tabacum* (tobacco) was only found in NEVL males (Figure 2).

## Discussion

This is the first study to survey taxa associated with an infectious disease vector applying an unbiased and comprehensive metagenomic approach. To ensure an unbiased description of the microbial community, the rationale chosen for this study included the extraction of total RNA and sequence-independent amplification. Total RNA was extracted from wild adult male and female *Lu. longipalpis* from an endemic (Posadas, Misiones) and a non-endemic (Lapinha Cave, Minas Gerais) VL location in Argentina and Brazil, respectively, and submitted to high-throughput pyrosequencing [44]. Given the high background level of vector sequences (∼85%), this approach proved to be very sensitive since it enabled the identification of taxa present in percentages up to 0.00036%. Moreover, as the different taxa were identified by homology to both rRNA and mRNA, the chosen approach was adequate for the objectives of this study.

The bacterial community identified in females from both locations and in NEVL males was distinct in every case. The only results in common with previous studies of gut microbiota from wild and laboratory reared female *Lu. longipalpis* and laboratory reared female *P. duboscqi* [18]–[20], [55], included the prevalence of Gram negative bacteria and the identification of *A. baumanni*, which in this study was found in NEVL males. Although previous reports established an essential basis for phlebotomine gut microbiota current knowledge, in these studies bacteria were identified using standard bacteriological methods. Consequently, those descriptions did not consider the remaining 99% of unculturable environmental microbes [56]. Hence the differences with this study, which applied a culture independent unbiased high-throughput approach that bypassed cloning of environmental DNA.

Interestingly and in accordance with results from this study, in previous reports the proportion of bacteria isolated from wild dipterans has been low. Studies on the midgut microbiota of wild mosquitoes, isolated bacteria from less than 50% of the specimens and the numbers of bacteria varied between individuals [57]–[58]. In a more recent study which used culture dependent and independent screening of field-collected *Anopheles*, bacteria were found in 15% of the mosquitoes, few of the mosquitoes harboured more than one bacterial species and only one species was found in more than one mosquito [23].

Only one bacterial type was found in EVL females (SS1), which corresponded to an unculturable bacterium originally isolated from cow faeces (Figure 2). Furthermore, a sequence match against RDP [52] indicated high similarity with *Alistipes* sp., a Gram negative anaerobic bacteria found in human faeces (Figure 2). Five bacterial species were found in NEVL females (PP1), four of which were Gram positive (Figure 2). Of these species, *R. pickettii*, *A. flavithermus*, *G. kaustophilus* and *S. coelicolor*, were originally isolated from contaminated lake sediment, waste water [59], deep-sea sediment [60] and soil [61], respectively. Interestingly, *A. flavithermus* and *G. kaustophilus* are thermophilic. Even though *R. pickettii* 12D was originally isolated from contaminated lake sediment, it is a ubiquitous microorganism found in water and soil [62] and is emerging as an opportunistic pathogen found in a wide variety of clinical samples [63]. *P. acnes* [64] is a universal inhabitant of human skin and is found at high population densities on the fat-rich areas of the face, scalp and upper trunk [65]. Four bacterial types were found in NEVL males (PP2), 50% of which were Gram negative (Figure 2). One of these bacterial types was the multidrug-resistant *A. baumannii* [66], which is recovered from natural environments and has emerged as an important opportunistic pathogen worldwide [67]. Another of the bacterial types corresponded to uncultured *Veillonella* sp. isolated from human skin [68]. The other two bacteria were uncultured bacterial types originally recovered from environmental samples. In one case, BLASTN analysis indicated homology both to an uncultured bacterium from a water sample (Atlantic Ocean) and to *Leifsonia xyli*, a sugar-cane pathogen [69]. The other bacterial sequence corresponded to a proteobacterium clone isolated from wetland soil [70].

In summary, bacteria identified in this study are ubiquitous in the diverse environments these sandflies frequent (faeces, soil, water, sediment, plants, human skin) and which were present in both sampling sites (Figure 1, Table 1). Hence, possibly they were indicative of the behavioural patterns and feeding habits of these sandflies and are probably part of their transient microbiota. However, more in depth research is required to determine these interactions.

Four different species of fungi were found in NEVL *Lu. longipalpis* (PP2 and PP1), which differed between males and females (Figure 2). In previous reports for *P. papatasi* and *P. tobbi*, mycoses with a high incidence rate were found in the guts and malpighian tubes of wild specimens. Similar fungi cultured from guts of laboratory reared *P. papatasi* were identified as *A. sclerotiorum* and *S. cerevisiae* [25]. Microsporidians, which are highly pathogenic for some insects [71], have also been found parasitising neotropical sandflies [32], [72]. The two species identified in this study in NEVL females were *P. conglomerata* [73], a plant pathogen (mildew), and *C. bertholletiae* [74], a common soil fungus and a rare cause of zygomycosis in humans. On the other hand, the species found in NEVL males were *M. verticillata* [75], a genus commonly found in soil and a zygomycete which also causes zygomycosis in animals, and *T. irritans* [76], which belongs to a genus of foliar pathogens [77]. Given the very high vegetation density in the Lapinha Cave area (Figure 1, Table 1), a possible scenario is that plant pathogenic spores adhered to the sandflies' hairy surface during sugar-feeding on infected plants. This suggested *Lu. longipalpis* has a putative capacity of casual dispersal of plant pathogens, among others (see below). In conclusion, fungi identified in this study are found ubiquitously in the environments frequented by sandflies (plants and soil), which were abundant in the sampling site (Lapinha Cave), and so were probably indicative of their sugar-feeding habits and behavioural patterns.

Protist sequences were only found in EVL male and female specimens, of which the vast majority were found in males (Figure 2). Nearly 90% of the identified apicomplexans corresponded to coccidians (genera *Cryptosporidium*, *Eimeria*, *Sarcocystis* and *Besnoitia*, 88.7%) and the rest to gregarines (genera *Ascogregarina* and *Psychodiella*, 7%) and haemosporidians (*Plasmodium* spp., 4.3%). The absence of leishmanial sequences was not unexpected considering the rate of infection of sandflies with *Leishmania* is generally very low (0.01–1%) [78], even in endemic areas [5].

Gregarines have been reported in over 20 species of sandflies and *Ascogregarina* spp. have only been described in mosquitoes [79]. Given *A. taiwanensis* sequences were found in EVL males in this study, this could indicate that the parasite also infects *Lu. longipalpis*. Genus *Psychodiella* comprises 3 species with host specificity to phlebotomine sandflies: *P. chagasi*, *P. saraviae* and *P. mackiei* [35], [79]. In the New World, only *P. chagasi* and *P. saraviae* have been found parasitising *Lutzomyia* spp. and *P. chagasi* seems to infect a large range of neotropical species [33]–[36]. In this study, *P. chagasi* sequences were found in EVL males. The exact pathology caused by gregarines is unknown, but in *Lu. longipalpis* the parasite can reduce longevity and egg production and the level of parasitaemia can reach over 80% in laboratory colonies [80]. Notwithstanding, the use of *P. chagasi* as a control method in the field has not been considered efficient because the parasite seems to have a limited range and a minimal effect on sandfly biology under natural conditions [81]. The fact that in this study *P. chagasi* was found in randomly caught wild specimens, suggested it could be a more efficient control method under natural conditions than what was previously reported.

The free-living oocyst stage of coccidians is discharged by infected animals through their faeces. Sandflies are found around human habitations and breed in specific organic wastes, exploiting the accumulation of organic matter produced by domestic animals and poor sanitary conditions such as faeces, manure, rodent burrows and leaf litter [5]. Since the EVL sampling site (Posadas) was a worst case-scenario homestead which included dense vegetation, various domestic animals and abundant organic matter (Figure 1, Table 1), this could account for the presence of these parasites in EVL males and females. On the other hand, female sandflies suck blood from different animal species including humans, bovines, pigs, equines, dogs, opossums, birds, various rodents and reptiles [5], [82] and, additionally, some *Plasmodium* spp. that parasitise lizards have been found in sandflies [37]. In the field it is common to see lek-like aggregations of males and females assembled on or near hosts where blood feeding and mating occur [83]–[84]. This behaviour could account for the presence of haemosporidian sequences (blood borne parasites) in EVL males. Nevertheless, as these sequences were found only in males, this suggested males, and not females, would be the primary source of *Plasmodium* spp. Furthermore, *P. falciparum* was recently identified by PCR in faecal samples from gorillas [85] and considering the EVL sampling site had a significant amount of organic matter (Figure 1, Table 1), it is highly feasible that males acquired these microorganisms from human faeces. Alternatively, EVL males could have acquired these microorganisms by contact with other vectors bearing *P. falciparum* (*i.e.*, *Anopheles* spp.) during transportation. In any case, these results suggest that, due to their behavioural patterns, *Lu. longipalpis* could be implicated in the casual dispersal of parasites of medical and veterinary importance.

Human sequences were found in males and females from both locations, whereas chicken sequences were found in NEVL females and EVL males and lizard sequences were only found in NEVL females (Figures 1 and 2). *Lu. longipalpis* is ubiquitous in dwellings where sanitary conditions are poor and domestic animals, such as dogs, chickens and pigs, are kept in and around the houses. In this kind of environment, the sandfly tends to congregate at outdoor sites, including animal sheds, where leks easily form on abundant, stationary hosts [7], [84]. In this context, as previously mentioned, the EVL location (Posadas) was a worst case-scenario homestead that kept dogs, chickens and a cat, had dense vegetation and a nearby spring (Figure 1, Table 1). In the NEVL location (Lapinha Cave), a chicken was kept to attract sandflies and as a source of food (Figure 1, Table 1). Furthermore, 35 species of lizards can be found in the Minas Gerais region [86]. As female sandflies blood feed on different animal species such as birds, reptiles and humans [5], [82], the presence of these sequences in females was not unexpected. Contrariwise, it was unexpected to find human and chicken sequences associated with males. Nonetheless, this could be due to their previously mentioned behavioural patterns of aggregation and courtship, where male sandflies are often seen over the host where they form leks, attracting females for a blood meal and increasing their chance for mating [83]–[84]. Alternatively, the trap itself was another area of close contact between male and female sandflies and with other potential vectors of medical importance. Consequently, males could have acquired these sequences by contact during transportation.

A total of ten different plant species were identified in males and females from both locations. *Capsicum annuum* (bell pepper), *Elaeis guineensis* (African oil palm) and *Solanum tuberosum* (potato) were identified in three of the four *Lu. longipalpis* samples. *Juglans hindsii* (Northern California walnut) was found in both EVL samples and *Artemisia annua* (sweet wormwood) was found in both male samples. *Rhapidophyllum hystrix* (needle palm) and *Brassica napus* (rapeseed) were only found in EVL females and *Vitis vinifera* (grapevine) was only found in EVL males. *Oryza sativa* (rice) and *Nicotiana tabacum* (tobacco) were only found in NEVL females and males, respectively (Figure 2). As adults from both sexes feed on sugars from different plant sources [12], this diversity could be indicative of the different feeding preferences and/or food source availability. Moreover, as sugar meals are not composed primarily by cells, plant RNA could also have originated from other sources. Namely, pollen dispersed by wind could have adhered to the sandflies' hairy surface or, alternatively, these vectors could be casual pollinators during sugar-feeding.

For a more comprehensive understanding of these results, a few limitations of the chosen approach should be considered. In the first place, homology searches are circumscribed to the number and quality of sequences in the databases at the time of analysis. The relatively high number of sequences which showed no significant hits (∼14%) was a clear indication of this. Moreover, if the query corresponds to a given organism that has not yet been sequenced, the hit will probably coincide with the most related organism found in the database. Notwithstanding and given this situation, the results from the homology search will provide a close approximation to the real case-scenario. Another aspect is that, similarly to a previous study [48] and in order to obtain as much environmental data as possible, specimens were neither surface cleaned nor dissected to extract their guts. As they were not surface cleaned, some (or all) of the identified taxa could have been surface contaminants, acquired during transportation by contact with other captured species, *i.e.* hymenopterans, lepidopterans and mosquitoes, or during manipulation in the lab. In the latter case, even though samples were manipulated with extreme care, this was still a potential source of contamination. Nevertheless, if contamination occurred during manipulation, it was plausible to expect the same contaminating species in males and females from the same location (when specimens were identified and separated according to sex) or from both locations (when total RNA was extracted). The only species present in all four samples was *Homo sapiens* and, consequently, contamination during manipulation was a possibility for these reads. Notwithstanding, as some biological control agents act by surface contact, such as *Beauveria bassiana* [39], [87], and since the ultimate goal of this study was to identify possible biological control agents for this neglected infectious disease vector, had the specimens been surface cleaned, this information could have been lost together with other valuable environmental data. On the other hand, as the gut was not separated from the rest of the specimen and, consequently, they were not analysed independently, it was not possible to classify the observed taxa in putative surface contaminants and gut inhabitants, among others. Therefore, the possible role of putative permanent gut residents could not be inferred, such as influence on the insect development cycle or on the parasite transmission ability. Nevertheless, even a careful extraction process would not preclude the possibility of cross-contamination between the gut and the rest of the specimen and/or loss of information. In this sense, the chosen approach ensured that no data was lost and, notwithstanding the aforementioned limitations, enabled the identification of taxa that could putatively influence sandfly development and which have become the target of ongoing studies to determine their significance and location in the sandfly.

Finally, the diversity of bacterial, fungal, protist, plant and metazoan sequences found in this study in wild adult *Lu. longipalpis* from endemic and non-endemic locations, mostly confirmed their feeding habits and behavioural patterns. Nevertheless, it also suggested that these vectors could possibly be a chance source of dispersal of various animal and plant diseases, such as coccidiosis and malaria. This is particularly significant since the geographical distribution of this vector is undoubtedly expanding [7]. The fact that RNA was obtained from these animal and plant pathogens would indicate that they were biologically active, but this cannot be determined with the present results and further studies must be performed to establish the significance of these findings. The identification of gregarines in wild *Lu. longipalpis* specimens could indicate that these parasites are a more efficient control method under natural conditions than what was previously suggested [81]. This is specially meaningful as studies on biological control of phlebotomines are still scarce and its practical application seems to be limited to the adult VL vector stage [16]. The employment of biolarvicides in the field is difficult due to the diversity of habitats in which this vector can reproduce and evidence that *Lu. longipalpis* larvae appear to be thinly dispersed and not concentrated in any particular microhabitat [17]. Nevertheless, as the number of samples analysed in this study was limited, a greater number of specimens must be studied to establish the significance of these results. Current studies are underway to analyse the presence and establish the significance of the taxa found in this study in a greater number of adult male and female *Lu. longipalpis* samples from endemic and non-endemic locations. A particular emphasis is being given to those taxa implicated in the biological control of this vector and to the etiologic agents of animal and plant diseases.

## Supporting Information

Table S1 **This table lists all the reads that showed significant hits in the homology database searches (1e-50 cutoff E-value).** Reads are grouped according to the sample they were identified in (PP1, SS1, PP2 or SS2). The information provided for each read includes: read accession number (number assigned to each read by NCBI SRA), read number (number assigned to each read when the samples were sequenced), hit accession number (of the hit with the highest E-value), a brief description of the hit and its E-value as indicated by the homology search.
(XLS)
